# Association between genetic variants in TREM1, CXCL10, IL4, CXCL8 and TLR7 genes with the occurrence of congenital Zika syndrome and severe microcephaly

**DOI:** 10.1038/s41598-023-30342-3

**Published:** 2023-03-01

**Authors:** Camilla Natália Oliveira Santos, Lucas Sousa Magalhães, Adriana Barbosa de Lima Fonseca, Ana Jovina Barreto Bispo, Roseane Lima Santos Porto, Juliana Cardoso Alves, Cliomar Alves dos Santos, Jaira Vanessa de Carvalho, Angela Maria da Silva, Mauro Martins Teixeira, Roque Pacheco de Almeida, Priscila Lima dos Santos, Amélia Ribeiro de Jesus

**Affiliations:** 1 Immunology and Molecular Biology Laboratory and Graduate Program in Health Sciences, University Hospital of Federal University of Sergipe, Aracaju, Brazil; 2 Sector of Parasitology and Pathology, Biological and Health Sciences Institute, Federal University of Alagoas, Maceió, Brazil; 3 Pediatric Division of University Hospital of the Federal University of Sergipe, Aracaju, Brazil; 4 Central Public Health Laboratory of Sergipe, Health Foundation Parreiras Horta, Aracaju, Brazil; 5 Department of Medicine of University Hospital, Federal University of Sergipe, Aracaju, Brazil; 6 Federal University of Minas Gerais, Belo Horizonte, Brazil

**Keywords:** Immunogenetics, Infectious diseases, Viral infection

## Abstract

Congenital Zika syndrome (CZS) is a cluster of malformations induced by Zika virus (ZIKV) infection and the underline mechanisms involved in its occurrence are yet not fully understood. Along with epidemiological and environmental factors, the genetic host factors are suggested as important to the CZS occurrence and development, however, few studies have evaluated this. This study enrolled a total of 245 individuals in a case–control association study compound a cohort of high specific interest constituted by 75 mothers who had delivered CZS infants, their 76 infants, and 47 mothers that had delivered healthy infants, and their 47 infants. Sixteen single-nucleotide polymorphisms on TREM1, CXCL10, IL4, CXCL8, TLR3, TLR7, IFNR1, CXCR1, IL10, CCR2 and CCR5 genes were genotyped to investigate their association as risk factors to CZS. The results show an association between C allele at TREM1 rs2234246 and C allele at IL4 rs224325 in mothers infected with ZIKV during pregnancy, with the increased susceptibility to CZS occurrence in their infants and the SNP CXCL8 rs4073 and the G allele at CXCL10 rs4508917 with presence of CZS microcephaly in the infants. Furthermore, the T allele at CXCL8 rs4073 and TRL7 rs179008 SNPs were associated with the severity of microcephaly in children with CZS. These results suggest that these polymorphisms in genes of innate immune responses addressed here are associated to increased risk of occurrence and severity of CZS in pregnant mothers infected with ZIKV and their CZS infants.

## Introduction

Congenital Zika syndrome (CZS) is a cluster of neurological congenital malformations, especially microcephaly, that can occur as consequence of vertical transmission of Zika virus (ZIKV) from mothers infected during pregnancy to their fetus^1,2^. The human infection by this arthropod-borne flavivirus is mostly transmitted by *Aedes* mosquitoes, although there are other forms of transmission^3.^ Also, infections are usually asymptomatic or trigger mild disease^4,5^, however severe disease, represented mostly by neurologic disorders, as Guillain–Barré syndrome and the CZS^1,6,7^, can occur and are the most important clinical outcome o-f ZIKV infection. CZS occurs only in a small percent of infections during pregnancy^1,8^. The rate of occurrence of malformation resulting from CZS seems to vary according to geographical location, the gestational age and immune factors. These observations and the knowledge that the disease caused by ZIKV infection is multifactorial, in which environmental and epigenetic determinants, characteristics of the pathogen and the host, influence both the occurrence of the disease and its clinical presentation, suggests that host genetics might be an important factor to this outcome, although the genes involved still need to be better understood^9,10^.

Among other factors, as the ones linked to cellular maturity and placental permeability, the immune response developed during maternal–fetal transmission of ZIKV leading to malformations is not completely elucidated and there are difficulties in understanding which factors contribute to the worsening of clinical cases^9,10^. Previous studies have already demonstrated the participation of several genes or proteins of innate and adaptive immune responses as markers of ZIKV infection and CZS^11–13^, but in the context of disease it is difficult to know whether the seen phenotype is cause or consequence of disease.

Previous studies suggested that CZS may be influenced by genetic and/or epigenetic differences^10^ and shown association between variations in adenylate cyclases^14^, NOS2, TNFα^15^, Interferon-λ^16^ and TP53^17^ and de occurrence of CZS. Our group has shown an association between the single nucleotide polymorphism (SNP) in rs3775291 at TLR3, which has been previously shown to affect 50% of the function of this receptor to trigger to type I interferons antiviral responses^18^, in mothers infected by ZIKV during pregnancy and the CZS occurrence^19^. Additionally, this study also shown an association between the SNP rs1799964 at TNFα gene in the CZS babies, a low producer allele, with severe microcephaly^19^, reinforcing the relevance of genetic factors to ZIKV pathogenesis.

Here, using hypothesis-driven candidate genes and their previous report in the ZIKV pathogenesis or their function against viral infections or other infection disease, we tested SNPs in TREM1^20–22^, CXCL10^11,23^, IL4^24,25^, CXCL8^26^, TLR3^19^, TLR7^27–29^, IFNR1^30,31^, CXCR1^32^, IL10^33–35^, CCR2 and CCR5^36^ genes to investigate their association to CZS, in a cohort of mothers who had delivered CZS infants, their infants and healthy donors.

## Methods

### Characterization of the clinical cohort

Seventy-five women who gave birth to babies with CZS (M-MICRO) from August 2015 to March 2017 and their seventy-six CZS babies (C-MICRO) were enrolled in this study and compound de case group. One of these mothers had monozygotic twins with CZS. The CZS children included here are attended at the pediatric service of the University Hospital of the Federal University of Sergipe, SE, Brazil, that is the reference center for treatment and follow-up of CZS babies in the State and were obtained by convenience and in a consecutive way. The children with CZS were followed over time by a multidisciplinary team, including pediatricians, neuropediatricians, physiotherapists, ophthalmologists, phonoaudiologists, among others. The medical team examining clinical history and performed clinical evaluation to confirm the absence of other complications during pregnancy, besides the ZIKV infection, as well as the presence of neurological congenital damage and other malformations that configure the CZS. According to the guidelines of the Brazilian Ministry of Health in Brazil, which follows the World Health Organization’s recommendations, the CZS babies were classified with severe microcephaly (CSM—more than 3 standard deviations below the mean for gestational age and sex) and microcephaly (CM—more than 2 standard deviations below the mean for gestational age and sex) at birth^37^. Other infections associated with neurological injury (STORCH) were discarded by serological tests. The control group was composed of 47 mothers (M-ZIKVexp) and their healthy babies (C-CT), who were matched to the CZS infants’ group by place of residence and month of birth (about 2 months of interval). All children were born in the municipalities of Sergipe state, northeast region of Brazil and all mothers lived in this same state as well. All Epidemiological data were obtained by a survey questionnaire developed by the research team. For serological assays we used Anti-Zika Virus ELISA (IgM) and Anti-Zika Virus ELISA (IgG) (Euroimmun, Medizinische Labordiagnostika AG), following manufacturer's instructions.

### Genetic analysis

Whole blood samples were collected from all patients from which serum, plasm or DNA samples were obtained. Genomic DNA was extracted using PureLink® Genomic DNA Mini Kit (Invitrogem™) according to the manufacturer’s recommendations. After extraction, the DNA concentration was determined with a NanoDrop™ Lite (Thermo Scientific, Wilmington, EUA) and stored at 80 °C until use.

Sixteen SNPs from candidate genes were selected, based on their previous association with viral or other infectious diseases and the gene importance in the immune response against viruses or plausible contribution to by ZIKV diseases (Supplementary Table S1). The SNPs TREM1 rs2234246, CXCL10 rs4508917, IL4 rs2243250, CXCL8 rs4073, TLR3 rs3775290, TLR7 rs179008, IFNR1 rs2234711, CXCR1 rs2854386, IL10 rs1800871/ rs1800872/ rs1800896, CCR2 rs1799864 and CCR5 rs1800023/ rs1800024/ rs1799987/ rs1799987 were genotyped using TaqMan® probes by qPCR using 7500 Real-Time PCR (Applied Biosystems) following manufacturer’s instructions. Information about the polymorphisms evaluated are found in the Supplementary Table S1.

### Statistical analysis

The Hard-Weinberg equilibrium (HWE) test was performed using GENEPOP (version 4.2). Categorical variables were compared between the groups by Chi-square test or Fisher’s Exact Test using GraphPad Prism (version 5.0). The associations between the occurrence of CZS and the SNPs were assessed by comparing case and control groups through a univariate logistic regression analysis using R software (version 3.4) with the package “SNPassoc”. To the descriptive analysis between groups, quantitative variables were tested through the Shapiro–Wilk test to verify their normality and according to the distribution found, Student’s t test or Mann–Whitney U test were utilized. The results were evaluated considering a confidence interval (CI) of 95% and the values ​​were considered statistically significant when *p* < 0.05.

### Ethics approval and consent to participate

This study was approved by the local Research Ethical Committee of the Federal University of Sergipe (advice number 1.486.302). All blood donors or their legal guardian gave written informed consent for their participation in the study.

## Results

### Clinical and epidemiological characteristics from the cohort

The characteristics of the patients included in this study are shown in Table 1. The age range in M-MICRO and M-ZIKVexp groups was 14–40 years, with an average of 25.4 and 24.3, respectively. Regarding to the presence of symptoms, higher frequency of mothers who composed the M-MICRO were symptomatic (76%), as compared to the M-ZIKVexp group (25.5%) (*p* ≤ 0.0001). The most frequent symptoms reported were axanthema, fever, and arthralgia, with 80.7%, 61.4% and 59.6% for the M-MICRO group and 50%, 66.7% and 58.3% for the ZIKVexp group, respectively. Regarding to the period of occurrence of symptoms, in most mothers in the M-MICRO group (50%), symptoms occurred during the first trimester of pregnancy, followed by 36.8% in the second trimester and 13.2% in the third trimester. In the M-ZIKVexp group, the trimester with the highest occurrence of symptoms was the second (50%), followed by the first trimester (33.3%) and the third trimester (16.7%).

**Table 1:** Clinical-epidemiological characteristics of the cohort from Northeast Brazil.

Variables	Case group	Control group	OR (95% CI)	*p*
	M-MICRO	M-ZIKVexp		
Total no	(*n* = 75)	(*n* = 47)		
Age	25.4 (14–40)	24.3 (14–40)		0.312
Symptoms occurrence				
Asymptomatic	18 (24)	35 (74.5)	9.23 (3.95–20.71)	**< 0.0001**
Symptomatic	57 (76)	12 (25.5)		
Symptomatology during pregnancy (% in all symptomatic)				
Fever	35 (61.4)	8 (66.7)	0.79 (0.24–2.77)	> 0.999
Arthralgia	34 (59.6)	7 (58.3)	1.05 (0.32–3.68)	> 0.999
Exanthema	46 (80.7)	6 (50.0)	4.18 (1.18–13.50)	0.058
Conjunctivitis	4 (7.0)	0		> 0.999
Myalgia	28 (49.1)	6 (50.0)	0.96 (0.27–3.38)	> 0.999
Retro-orbital pain	20 (35.1)	3 (25.0)	1.62 (0.43–6.02)	0.737
Lymphadenopathy	7 (12.3)	2 (16.7)	0.70 (0.14–3.74)	0.650
Pruritus	7 (12.3)	0		0.340
Trimester of ZIKV infection symptoms occurrence				
1st	19/ 38 (50.0)	2/ 6 (33.3)		0.748
2nd	14/ 38 (36.8)	3/ 6 (50.0)		
3rd	5/ 38 (13.2)	1/ 6 (16.7)		
IgG ZIKV				
Positive	71 (94.7)	24 (51.1)		
Negative	0	20 (42.5)		
Borderline	4 (5.3)	3 (6.4)		
IgG DENV				
Positive	68 (90.7)			
Negative	4 (5.3)			
Borderline	3 (4.0)			

Variables	Case group	Control group	OR (95% CI)	*p*
	C-MICRO	C-CT		
Total no	(*n* = 76)	(*n* = 47)		
Sex (%)				
Female	41 (53.9)	17 (36.2)	2.06 (0.95–4.44)	0.064
Male	35 (46.0)	30 (63.8)		
CP classification at birth				
Age-appropriate	0	44 (100)		
Severe microcephaly**	42/ 60 (70.0)	0		
Microcephaly**	18/ 60 (30.0)	0		
Birth period	Jul. 2015–Mar. 2017	Apr. 2015–Jul. 2017		

M-MICRO, women who gave birth to infants with congenital Zika syndrome; M-ZIKVexp, mothers who living in ZIKV’s endemic areas who gave birth to healthy infants; C-MICRO, children with congenital Zika syndrome; C-CT, healthy children who were born of the mothers who living in ZIKV’s endemic areas; Symptomatic: report of two or more of the listed symptoms of ZIKV infection. Quantitative variables were compared between groups using Student’s t test or Mann–Whitney U test and are presented as mean minimum and maximum value; Categorical variables were compared between groups using Chi-squared test or Fisher’s exact test.

Bold indicates statistically significant.

**Individuals in this category may present other malformations in addition to microcephaly.

The C-MICRO group was composed of 53.9% of females and 46.0% of males, while the C-CT group was composed of 36.2% and 63.8% of females and males, respectively. Regarding the classification of CP at birth, most children (70%) in the C-MICRO group were born with severe microcephaly (CSM), while 30% of them were born with microcephaly (CM) according to the classification explained previously. In the C-CT group, all children were born with an adequate CP for their gestational age. There were no significant differences between the period of birth of children included in the case and control groups.

To better characterize the C-MICRO group, children with microcephaly (CM) and with severe microcephaly (CSM) were evaluated and described in Table 2. Significant differences were found between groups in cephalic perimeter at birth (*p* ≤ 0.0001), birth weight (*p* = 0.004) and length at birth (*p* = 0.006). The differences between the groups regarding the duration of pregnancy, presence of symptoms in mothers during pregnancy and the trimester of occurrence of symptoms related to ZIKV infection were not significant.

**Table 2:** Clinical-epidemiological variables in cases of CZS stratified by severity of microcephaly.

Variables	CSM** (n = 42)	CM** (*n* = 18)	*p*
Sex			
Female (%)	23 (54.8)	10 (55.6)	> 0.999
Male (%)	19 (45.2)	8 (44.4)	
Weight at birth(g)	*n* = 24	*n* = 5	
	2.57 (1.30–3.70)	3.18 (2.95–3.40)	**0.004**
Height at birth(cm)	*n* = 21	*n* = 5	
	45.2 (34.0–49.0)	48.4 (47.0–49.0)	**0.006**
Duration of pregnancy	*n* = 40	*n* = 18	
Pre-term	2 (5)	2 (11.1)	0.689
Term	36 (90)	15 (83.3)	
Post-term	2 (5)	1 (5.6)	
Symptoms of ZIKV infection in the mother	*n* = 42	*n* = 18	
Symptomatic (%)	34 (81.0)	11 (61.1)	0.118
Asymptomatic (%)	8 (19.0)	7 (38.9)	
Trimester of ZIKV infection symptoms occurrence during pregnancy	*n* = 24	*n* = 7	
1st	12 (50)	5 (71.4)	0.562
2nd	8 (33.3)	1 (14.3)	
3rd	4 (16.7)	1 (14.3)	
Cephalic perimeter at birth (cm)	28.25 (22.5–30.0)	31.0 (30.0–34.0)	**< 0.0001**

CSM, children with severe microcephaly due congenital Zika syndrome; CM, children with microcephaly due congenital Zika syndrome; The information about the trimester of ZIKV infection during pregnancy was not available or mothers responded that they did not remember; Pre-term: less than 259 days (37 weeks), term: 259–293 days (37–41 weeks), post-term: 294 days (42 weeks) or more; Quantitative variables were compared between groups using Student’s t test or Mann–Whitney U test and are presented as mean minimum and maximum value; Categorical variables were compared between groups using Chi-squared test or Fisher’s exact test.

Bold indicates statistically significant.

**Individuals in this category may present other malformations in addition to microcephaly.

### Association of SNPs with CZS and clinical characteristics

General information about SNPs analyzed in the present work are presented in Supplementary Table S1. The EHW test showed equilibrium deviation in the control population for the SNPs CXCL10 rs4508917, TLR7 rs179008 and IL10 rs1800896.

The C allele for the SNP rs2234246 in the TREM1 gene was more frequent and associated with mothers who gave birth to CZS babies (M-MICRO) and their babies (C-MICRO) when compared with mothers (M-ZIKVexp) and children (C-CT) control groups (Table 3). Furthermore, the C allele for the IL4 rs224325 gene was more frequent and associated with mothers who gave birth to CZS infants (M-MICRO) (Table 4). No significant differences were found among case and control infants for this SNP regarding allelic or genotypic frequencies in any of the analysis performed.

**Table 3:** Genotype and allelic frequency and distribution of TREM1 rs2234246 SNP and its association with CZS.

Gene/SNP	Case group	Control group	OR (95% CI)	*p*	Case group	Control group	OR (95% CI)	*p*
	M-MICRO	M-ZIKVexp			C-MICRO	C-CT		
TREM1 rs2234246	*n* = 73	*n* = 47			*n* = 76	*n* = 46		
TT	15 (20.5)^a^	13 (27.7)		**0.035**	19 (25.0)	21 (45.7)		0.060
CT	41 (56.2)	31 (65.9)	1.15 (0.48–2.76)		41 (53.9)	19 (41.3)	2.39 (1.04–5.44)	
CC	17 (23.3)	3 (6.4)	4.91 (1.17–20.62)		16 (21.1)	6 (13.0)	2.95 (0.96–9.08)	
TT	15 (20.5)	13 (27.7)	1.48 (0.63–3.48)	0.371	19 (25.0)	21 (45.7)	2.52 (1.16–5.49)	**0.019**
CT + CC	58 (79.5)	34 (72.3)			57 (75.0)	25 (54.3)		
TT + CT	56 (76.7)	44 (93.6)	4.45 (1.23–16.16)	**0.010**	60 (78.9)	40 (87.0)	1.78 (0.64–4.93)	0.255
CC	17 (23.3)	3 (6.4)			16 (21.1)	6 (13.0)		
C	75 (51.4)	37 (39.4)	1.62 (0.95–2.72)	0.084^b^	73 (48.0)	32 (34.0)	1.79 (1.04–3.06)	**0.034**^b^
T	71 (48.6)	57 (60.6)			79 (52.0)	62 (66.0)		
CC	17 (23.3)	3 (6.4)	4.91 (1.29–18.05)	**0.031**^b^	16 (21.0)	6 (12.8)	2.94 (1.02–9.66)	0.066^b^
TT	15 (20.5)	13 (27.7)			19 (25.0)	21 (44.7)		
log-Additive	73	47	1.87 (1.02–3.45)	**0.039**	76	46	1.85 (1.06–3.22)	**0.026**

M-MICRO, women who gave birth to infants with congenital Zika syndrome; M-ZIKVexp, mothers who living in ZIKV’s endemic areas who gave birth to healthy infants; C-MICRO, children with congenital Zika syndrome; C-CT, healthy children who were born of the mothers who living in ZIKV’s endemic areas.

Bold indicates statistically significant.

^a^(%) number of the subjects with the specified allele or genotype.

Results of univariate logistic regression models and ^b^Fisher’s exact test.

**Table 4:** Genotype and allelic frequency and distribution of CXCL10 rs4508917, IL4 rs2243250 and CXCL8 rs4073 SNP and their association with CZS.

Gene/SNP	Case group	Control group	OR (95% CI)	*p*	Case group	Control group	OR (95% CI)	*p*
	M-MICRO	M-ZIKVexp			C-MICRO	C-CT		
CXCL10 rs4508917	*n* = 73	*n* = 47			*n* = 76	*n* = 47		
AA	38 (52.1)^a^	29 (61.7)		0.538	37 (48.7)	32 (68.1)		0.089
AG	27 (37.0)	13 (27.7)	1.59 (0.70–3.60)		29 (38.2)	10 (21.3)	2.51 (1.06–5.93)	
GG	8 (11.0)	5 (10.6)	1.22 (0.36–4.13)		10 (13.2)	5 (10.6)	1.73 (0.54–5.59)	
AA	38 (52.1)	29 (61.7)	1.48 (0.70–3.13)	0.297	37 (48.7)	32 (68.1)	2.25 (1.05–4.81)	**0.033**
AG + GG	35 (47.9)	18 (38.3)			39 (51.3)	15 (31.9)		
AA + AG	65 (89.0)	42 (89.4)	1.03 (0.32–3.37)	0.956	66 (86.8)	42 (89.4)	1.27 (0.41–3.98)	0.675
GG	8 (11.0)	5 (10.6)			10 (13.2)	5 (10.6)		
AA + GG	46 (63.0)	34 (72.3)	1.54 (0.69–3.40)	0.287	47 (61.8)	37 (78.7)	2.28 (0.99–5.28)	**0.046**
AG	27 (37.0)	13 (27.7)			29 (38.2)	10 (21.3)		
GG	8 (11.0)	5 (10.6)	1.22 (0.39–3.72)	> 0.999^b^	10 (13.2)	5 (10.6)	1.73 (0.53–4.96)	0.403^b^
AA	38 (52.0)	29 (61.7)			37 (48.7)	32 (68.1)		
log-Additive	73	47	1.24 (0.72–2.16)	0.431	76	47	1.60 (0.92–2.79)	0.086

IL4 rs2243250	*n* = 75	*n* = 47			*n* = 76	*n* = 47		
CC	40 (53.3)	18 (38.3)		0.154	40 (52.6)	18 (38.3)		0.259
TC	26 (34.7)	18 (38.3)	0.65 (0.29–1.47)		27 (35.5)	20 (42.6)	0.61 (0.27–1.36)	
TT	9 (12.0)	11 (23.4)	0.37 (0.13–1.04)		9 (11.8)	9 (19.1)	0.45 (0.15–1.32)	
CC	40 (53.3)	18 (38.3)	0.54 (0.26–1.14)	0.104	40 (52.6)	18 (38.3)	0.56 (0.27–1.17)	0.120
TC + TT	35 (46.7)	29 (61.7)			36 (47.4)	29 (61.7)		
CC + TC	66 (88.0)	36 (76.6)	0.45 (0.17–1.18)	0.102	67 (88.2)	38 (80.9)	0.57 (0.21–1.55)	0.270
TT	9 (12.0)	11 (23.4)			9 (11.8)	9 (19.1)		
C	106 (70.7)	54 (57.4)	1.78 (1.04–3.04)	**0.038**^b^	107 (70.4)	56 (59.6)	1.61 (0.94–2.74)	0.096^b^
T	44 (29.3)	40 (42.6)			45 (29.6)	38 (40.4)		
CC	40 (53.3)	18 (38.3)	2.71 (1.01–7.79)	0.065^b^	40 (52.6)	18 (38.3)	2.22 (0.74–6.73)	0.166^b^
TT	9 (12.0)	11 (23.4)			9 (11.8)	9 (19.2)		
log-Additive	75	47	0.61 (0.37–1.01)	0.054	76	47	0.66 (0.39–1.09)	0.104

CXCL8 rs4073	*n* = 73	*n* = 47			*n* = 76	*n* = 47		
AA	28 (38.4)	16 (34.0)			29 (38.2)	11 (23.4)		
AT	32 (43.8)	23 (48.9)	0.80 (0.35–1.80)	0.853	28 (36.8)	27 (57.4)	0.39 (0.16–0.94)	0.075
TT	13 (17.8)	8 (17.0)	0.93 (0.32–2.72)		19 (25.0)	9 (19.1)	0.80 (0.28–2.30)	
AA	28 (38.4)	16 (34.0)	0.83 (0.39–1.78)	0.631	29 (38.2)	11 (23.4)	0.50 (0.22–1.12)	0.085
AT + TT	45 (61.6)	31 (66.0)			47 (61.8)	36 (76.6)		
AA + AT	60 (82.2)	39 (83.0)	1.06 (0.40–2.78)	0.911	57 (75.0)	38 (80.9)	1.41 (0.58–3.44)	0.448
TT	13 (17.8)	8 (17.0)			19 (25.0)	9 (19.1)		
AA + TT	41 (56.2)	24 (51.1)	0.81 (0.39–1.70)	0.584	48 (63.2)	20 (42.6)	0.43 (0.21–0.91)	**0.025**
AT	32 (43.8)	23 (48.9)			28 (36.8)	27 (57.4)		
log-Additive	73	47	0.93 (0.56–1.56)	0.790	76	47	0.85 (0.52–1.39)	0.515

M-MICRO, women who gave birth to infants with congenital Zika syndrome; M-ZIKVexp, mothers who living in ZIKV’s endemic areas who gave birth to healthy infants; C-MICRO, children with congenital Zika syndrome; C-CT, healthy children who were born of the mothers who living in ZIKV’s endemic areas.

Bold indicates statistically significant.

^a^(%) number of the subjects with the specified allele or genotype.

Results of univariate logistic regression models and ^b^Fisher’s exact test.

The SNP rs4508917 in the CXCL10 gene was also associated with the occurrence of microcephaly when the C-MICRO group and the C-CT group were compared, showing a higher frequency and association of G allele in children with CZS. The SNP rs4073 in the CXCL8 gene was also associated with the occurrence of CZS. There were no significant differences in allele frequencies among case and control group of mothers regarding these SNPs (Table 4).

Stratifying the case group of children (C-MICRO) by the severity of microcephaly at birth, in children with severe microcephaly (CSM) and children with microcephaly (CM), the T allele in SNP rs4073 at CXCL8 gene and the T allele in SNP rs179008 at TRL7 was associated with severe microcephaly (Table 5).

**Table 5:** Association between CXCL8 rs4073 and TRL7 rs179008 allele frequencies with microcephaly severity.

Gene/SNP	Severity of microcephaly		Allele comparisons	OR	95% CI	*p*
	CSM (%) *n* = 42	CM (%) *n* = 18				
TREM1 rs2234246						
C	39 (46.4)	15 (41.7)	C vs. T	1.21	0.54–2.59	0.691
T	45 (53.6)	21 (58.3)				
CXCL10 rs4508917						
A	58 (69.0)	25 (69.4)	A vs. G	0.98	0.43–2.18	> 0.999
G	26 (31.0)	11 (30.6)				
IL4 rs2243250						
C	56 (66.7)	29 (80.6)	C vs. T	0.48	0.19–1.20	0.187
T	28 (33.3)	7 (19.4)				
CXCL8 rs4073						
T	40 (47.6)	10 (27.8)	T vs. A	2.36	1.00–5.51	**0.046**
A	44 (52.4)	26 (72.2)				
TLR3 rs3775290						
C	59 (70.2)	26 (72.2)	C vs. T	0.90	0.37–2.07	> 0.999
T	25 (29.8)	10 (27.8)				
TLR7 rs179008						
T	21 (25.0)	3 (8.3)	T vs. A	3.66	1.06–12.2	**0.046**
A	63 (75.0)	33 (91.7)				
IFNGR1 rs2234711						
A	43 (51.2)	18 (50.0)	A vs. G	1.04	0.46–2.35	> 0.999
G	41 (48.8)	18 (50.0)				
CXCR1 rs2854386						
C	74 (88.1)	33 (91.7)	C vs. G	0.67	0.18–2.60	0.752
G	10 (11.9)	3 (8.3)				
IL10 rs1800871						
A	34 (40.5)	17 (47.2)	A vs. G	0.76	0.33–1.73	0.548
G	50 (59.55)	19 (52.8)				
IL10 rs1800872						
T	34 (40.5)	17 (47.2)	T vs. C	0.76	0.33–1.73	0.548
C	50 (59.5)	19 (52.8)				
IL10 rs1800896						
T	38 (45.2)	17 (47.2)	T vs. C	0.92	0.41–2.09	0.844
C	46 (54.8)	19 (52.8)				
CCR5 rs1800023						
A	64 (76.2)	27 (75.0)	A vs. G	1.06	0.45–2.62	> 0.999
G	20 (23.8)	9 (25.0)				
CCR2 rs1799864						
A	13 (15.5)	8 (22.2)	A vs. G	0.64	0.24–1.74	0.433
G	71 (84.5)	28 (77.8)				
CCR5 rs1799987						
A	40 (47.6)	22 (61.1)	A vs. G	0.57	0.26–1.28	0.231
G	44 (52.4)	14 (38.9)				
CCR5 rs1800024						
C	70 (83.3)	28 (77.8)	C vs. T	1.42	0.53–3.66	0.454
T	14 (16.7)	8 (22.2)				
CCR5 rs1799988						
C	41 (48.8)	22 (61.1)	C vs. T	0.60	0.27–1.35	0.237
T	43 (51.2)	14 (38.9)				

(%) number of subjects with the specified allele.

CSM, children with severe microcephaly due congenital Zika syndrome; CM, children with microcephaly due congenital Zika syndrome; *p*, Fisher's exact test.

Bold indicates statistically significant.

There was no association between the case and control group for the other SNPs assessed (Supplementary Table S2) and the haplotype analyzed (data not shown).

## Discussion

Faced with the serious public health problem occurring from ZIKV infections, the search for an understanding of the host factors that can modulate infections, interfering with their outcome, is essential. In this study, TREM1 rs2234246 and IL4 rs224325 in mothers infected with ZIKV during pregnancy was found to be associated with the occurrence of CZS microcephaly and the SNP CXCL10 rs4508917 and CXCL8 rs4073 in their children with presence of CZS microcephaly. Furthermore, the CXCL8 rs4073 and TRL7 rs179008 SNPs were associated with the severity of microcephaly in children with CZS.

The Triggering Receptor Expressed on Myeloid Cells 1 (TREM-1) is a cell surface receptor that is constitutively expressed in different types of human cells^38–40^. Its activation plays a key role in amplifying and regulating the inflammatory response in the innate immune response^38^ and has been studied in different diseases^41,42^. The rs2234246 in the TREM1 gene, located in the 3’UTR region, is characterized by a C > T variation and is described as a functional polymorphism, regulating TREM1 expression levels, with the T allele associated with higher levels of both gene expression and plasma levels of the soluble form of TREM-1 (sTREM-1) in healthy individuals^22^.

The findings of the present study show the association of the C allele for SNP rs2234246 in TREM1 as a risk factor for CZS. Although the role of TREM-1 has been little studied in viral infections so far, studies demonstrate increased gene expression of the TREM1 during viral infections in vitro, suggesting activation of TREM-1 signaling by viruses^43,44^. In addition, it is suggested that TREM-1 can recognize molecular patterns associated with viral pathogens, such as Polyinosinic:polycytidylic acid (Poly I:C), a TLR3 receptor ligand and important receptor in the pathogenesis caused by ZIKV^45^ and that this recognition leads to the induction of pro-inflammatory cytokines important for the reduction of viral load^21,43^.

Therefore, the data presented here suggest that this genetic variation in the TREM1 gene in pregnant women may influence the risk of developing CZS in their infants, indicating that lower expression/activation of TREM-1 may affect the control of ZIKV infection, which reinforces the need for a better understanding of the role of TREM-1 in the pathogenesis of CZS. Corroborating this hypothesis, previous data from our group has shown an association between SNPs in TLR3 and TNFα involved in the decrease of antiviral immune response, with CZS occurrence and severity^19^. In contrast, higher levels of sTREM-1 have been described as indicative of poor prognosis in early stages of DENV arbovirus infection^46^, and recently, on the outcomes of SARS-CoV-2 infections^47^.

The rs224325 SNP in the IL4 gene is in the UTR5' region and promotes the exchange of C>T nucleotides (-590C/T) and was shown that this SNP is functional, and that the alternative T variant increases IL4 promoting activity^24^, increasing levels of this protein^48^. The influence of this SNP on the outcome of diseases has been studied, with emphasis on the evaluation of its functioning in the pathogenesis of neoplasms, and it has already been associated with the susceptibility of different types of cancer^49,50^. In the present study, the C allele for IL4 rs224325 was more frequent and associated with mothers who had children with microcephaly due to ZIKV infection, which, according to the function described for this SNP, may be associated with lower levels of IL-4^24^. It is possible that this gene variation helps to promote an imbalance between the Th1 and Th2 immune response. Recent study demonstrated that tissue damage in the placenta is induced by ZIKV^51^, but the modulation of tissue damage by the anti-inflammatory response still needs clarification. In contrast, higher levels of IL-4 were detected during the acute phase of ZIKV infection in a mother who gave birth to a child with CZS, when compared to mothers who gave birth to normal children^11^.

Several studies demonstrate a cytokine storm associated with children with CZS^10–13,52^. However, few studies evaluate genetic factors in this condition. In the infants evaluated here, an association was found between the presence of at least one G allele for the rs4508917 SNP in the CXCL10 gene and the occurrence of microcephaly. This SNP promotes a variation of A > G, with the G allele being correlated with increased expression of the chemokine of the same name^23^. The CXCL10 is pointed as the most promising biomarker for acute ZIKV infection due to its overexpression^53^. In agreement, overexpression of CXCL10 has been linked to virus-induced signaling of the type 2 interferon IFNγ pathway, although ZIKV seems to suppress the IFN-1 and IFN-3 pathway as an evasion mechanism^54^. This chemokine has already been associated with other neurological complications^55,56^. Higher levels of CXCL10 were found in children with congenital malformations born from mothers infected with ZIKV during pregnancy, when compared to those control children born normal from mothers who were also ZIKV positive during pregnancy^11^. In a complementary way, this same study also observed higher levels of CXCL10 in patients infected with ZIKV who developed neurological complications^11^.

To assess whether the SNPs evaluated in this study were associated with the severity of CZS microcephaly, the case group of children was stratified in the analysis. This apporach revealed significantly different frequencies in the rs4073 SNP in the CXCL8 gene, and the rs179008 in the TRL7 gene between the groups, associating these SNPs with the severity of microcephaly.

The CXCL8 gene is located on the short arm of chromosome 4 and encodes the protein of the same name, also known as IL-8, that crucially participating in several inflammatory processes^57^. The rs4073 SNP (-251A>T) is in the promoter region of the gene and is related to variations in gene expression and levels of the CXCL-8 chemokine, with the A allele being related to higher levels of CXCL-8^58,59^. In the groups studied in this research, there was a higher frequency of the T allele in children with more severe microcephaly, an allele functionally associated with lower production of this chemokine. Due to the critical role that this cytokine plays, this SNP has been associated with different diseases^58,60–62^.

The endosomal receptor TLR-7 recognizes single-stranded RNA and plays an important role in the recognition of viral pathogens by activating the innate immune response to produce type 1 IFN^29^. The SNP rs179008 (A>T) is an intragenic variation and promotes the exchange of glutamine by leucine, affecting the quantity and functionality of the protein, with the T allele linked to the lowest expression of TLR7 and the lowest expression of IFNλ^28^. This variant has been associated with viral infections, being linked to different prognoses^27,28,63,64^. In the present study, an association between the T allele and more severe microcephaly is demonstrated.

Therefore, considering the functionality of these SNPs and the function of their produced proteins, the association of the T allele in CXCL8 rs4073 and the T allele in TLR7 rs179008 with the severity of microcephaly suggests that the impairing antiviral defense might attenuate a resolutive inflammatory response and contribute to CNS damage. These data agree with previous findings of our group that show an association of microcephaly with low producer alleles in genes from the antiviral response^19^. In line with this hypothesis, the presence of the TT genotype for this SNP in the TLR7 gene was previously associated with susceptibility to Herpes simplex virus -1 infection and an increased risk of placental infections^27^. Furthermore, higher levels of CXCL-8 in cerebrospinal fluid were found in neonates without CZS who were born from mothers infected with ZIKV during pregnancy, when compared to those born with microcephaly by CZS, suggesting an important role of this chemokine in protection against CZS^26^. Therefore, it is possible that the lower antiviral defense in the fetus predisposes to ZIKV invasion of the CNS and may be an aggravating factor in the neurological picture triggered by congenital ZIKV infection.

Despite being genes involved in immune response to viral infections and implicated in susceptibility to infections, no differences were found in genotypic and allelic frequencies between the groups of this study, in the analyzed SNPs TLR3, IFNGR1, CXCR1, IL10, CCR2 and CCR5. This fact does not rule out the influence of these SNPs on the pathogenesis triggered by ZIKV, since the non-association may have occurred due to the limited number of the samples, affecting the power of the study. In addition, differences in the frequencies of the SNPs that configurate the associations seen in the present study are limited to this population and not determinant to stablish cause and effect relation. Nevertheless, we had the opportunity to study a cohort of high specific interest, what reinforce the importance of this study as well of replication studies.

Together, the findings described here suggest that maternal genetics may influence the risk of the occurrence of CZS and that the genetics of children affected with CZS are associated with the severity of the syndrome, suggesting that an impaired antiviral response is associated with the immunopathogenesis of congenital ZIKV infection, and placing the genes TREM1, IL4, CXCL10, CXCL8 and TLR7 as promising genes for future functional studies, for a better understanding of their respective roles in the occurrence of CZS.

## Supplementary Information

Supplementary Tables.

## Data Availability

All main data generated or analyzed during this study are included in this article. Additional information about the data and material collected during this study are available on reasonable request by contacting the corresponding author (Camilla Santos, camillanatallia@hotmail.com).
