# Supplementary material for: Association between genetic variants in TREM1, CXCL10, IL4, CXCL8 and TLR7 genes with the occurrence of congenital Zika syndrome and severe microcephaly
Source: Sci Rep. 2023 Mar 1;13:3466. doi: 10.1038/s41598-023-30342-3 (PMC9975867; doi:10.1038/s41598-023-30342-3)
Supplement: Supplementary file 1 — Supplementary Tables. [file 41598_2023_30342_MOESM1_ESM.pdf]

Association between genetic variants in TREM1, CXCL10, IL4, CXCL8 and TLR7 genes with the occurrence of congenital Zika syndrome and severe microcephaly

Authors

Camilla Natália Oliveira Santos[1], M.Sc.

Lucas Sousa Magalães[1,2], Ph.D.

Adriana Barbosa de Lima Fonseca[3], M.D. and Ph.D.

Ana Jovina Barreto Bispo[3], M.D. and Ph.D.

Roseane Lima Santos Porto[3], M.D. and M.Sc.

Juliana Cardoso Alves[1], M.Sc.

Cliomar Alves dos Santos[4], Ph.D.

Jaira Vanessa de Carvalho[5], MD.

Angela Maria da Silva[5], M.D.and Ph.D.

Mauro Martins Teixeira[6], Ph.D.

Roque Pacheco de Almeida[1,5], M.D. and Ph.D.

Priscila Lima dos Santos[1], Ph.D.

Amélia Ribeiro de Jesus[1,5]. M.D. and Ph.D.

Authors Affiliations

[1] Immunology and Molecular Biology Laboratory and Graduate Program in Health Science, University Hospital of Federal University of Sergipe, Aracaju, Brazil.

[2] Sector of Parasitology and Pathology, Biological and Health Sciences Institute, Federal University of Alagoas, Maceió, Brazil

[3] Pediatric Division of University Hospital of the Federal University of Sergipe, Aracaju, Brazil.

[4]Central Public Health Laboratory of Sergipe, Health Foundation Parreiras Horta, Aracaju, Brazil.

[5] Department of Medicine of University Hospital, Federal University of Sergipe, Aracaju, Brazil

[6]Immunopharmacology, Federal University of Minas Gerais, Belo Horizonte, Minas Gerais, Brazil

Corresponding author

Camilla Natália Oliveira Santos. E-mail address: camillanatallia@hotmail.com.

Supplementary Table S1. Identification and general information of the single nucleotide polymorphisms (SNPs) assessed

Gene	SNP ID	Chromosome / Gene location	SNP type	Alternate Allele Frequency*	Alternate Allele Frequency**	Commercial SNP *Assay* code
TREM1	rs2234246	Chr.6/ 3' UTR	Transition Substitution	T= 50%	T = 43%	C___1165058_20
CXCL10	rs4508917	Chr.4/ Intron	Transition Substitution	G = 32%	G = 29%	C____497053_10
IL4	rs2243250	Chr.5/ Upstream	Transition Substitution	T = 29%	T = 34%	C__16176216_10
CXCL8	rs4073	Chr.4/ Upstream	Transversion Substitution	T = 51%	T = 46%	C__11748116_10
TLR3	rs3775290	Chr.4/ Intragenic	Transition Substitution	T = 28%	T = 27%	C__11785995_20
TLR7	rs179008	Chr.X/ Intragenic	Transition Substitution	T = 18%	T = 18%	C___2259574_10
IFNR1	rs2234711	Chr.6/ 5' UTR	Transition Substitution	G = 44%	G = 43%	C__11693991_10
CXCR1	rs2854386	Chr.2/ Downstream	Transversion Substitution	C = 89%	C = 86%	C__15826161_10
IL10	rs1800871	Chr.1/ Upstream	Transition Substitution	G = 67%	G = 69%	C___1747362_10
	rs1800872	Chr.1/ Upstream	Transversion Substitution	G = 67%	G = 67%	C___1747363_10
	rs1800896	Chr.1/ Intragenic	Transition Substitution	C = 35%	C = 40%	C___1747360_10
CCR2	rs1799864	Chr.3/ Intragenic	Transition Substitution	A = 13%	A = 12%	C__64650303_10
CCR5	rs1800023	Chr.3/ Intron	Transition Substitution	G = 35%	G = 30%	C___9698604_20
	rs1799987	Chr.3/ Intron	Transition Substitution	G = 47%	G = 49%	C__11988176_10
	rs1800024	Chr.3/ Intron	Transition Substitution	T = 14%	T = 13%	C__64650310_10
	rs1799988	Chr.3/ Intron	Transition Substitution	T = 47%	T = 49%	C__11988170_20

Gene, SNP identification, chromosomal location and gene region, type, alternate allele Frequency in *Brazilian population (ABraOM database) and **global (gnomAD database), and TaqMan® probes assay used in this study. Available at:<https://abraom.ib.usp.br/>, <https://gnomad.broadinstitute.org/>, <https://www.thermofisher.com/br/en/home.html>.

Supplementary Table S2. Frequency and distribution of genotypes of the SNP assessed

		Case group M-MICRO	Control group M-ZIKVexp	OR (95%CI)	*p*-Value	Case group C-MICRO	Control group C-CT	OR (95%CI)	*p*-Value
		n = 73	n = 47			n = 76	n = 47		
TLR-3 rs3775290	CC	43 (58.9)[a]	23 (48.9)			39 (51.3)	23 (48.9)		
	CT	26 (35.6)	19 (40.4)	0.73 (034 - 1.59)	0.431	33 (43.4)	17 (36.2)	1.14 (0.52 - 2.50)	0.190
	TT	4 (5.5)	5 (10.6)	0.43 (0.10 - 1.75)		4 (5.3)	7 (14.9)	0.34 (0.09 - 1.28)	
	log-Additive	73	47	0.69 (0.38 - 1.23)	0.202	76	47	0.75 (0.43 - 1.32)	0.320
		n = 73	n = 47			n = 76	n = 47		
TLR-7 rs179008	AA	51 (69.9)	31 (66.0)			57 (75.0)	37 (78.7)		
	AT	19 (26.0)	14 (29.8)	0.82 (0.36 - 1.88)	0.899	12 (15.8)	4 (8.5)	1.95 (0.58 - 6.50)	0.438
	TT	3 (4.1)	2 (4.3)	0.91 (0.14 - 5.76)		7 (9.2)	6 (12.8)	0.76 (0.24 - 2.43)	
	log-Additive	73	47	0.88 (0.46 - 1.68)	0.699	76	47	1.00 (0.58 - 1.74)	0.989
		n = 73	n = 47			n = 76	n = 47		
IFNGR-1 rs2234711	AA	19 (26.0)	12 (25.5)			19 (25.0)	11 (23.4)		
	AG	39 (53.4)	24 (51.1)	1.03 (0.42 - 2.48)	0.932	42 (55.3)	26 (55.3)	0.94 (0.38 - 2.28)	0.968
	GG	15 (20.5)	11 (23.4)	0.86 (0.30 - 2.49)		15 (19.7)	10 (21.3)	0.87 (0.29 - 2.59)	
	log-Additive	73	47	0.93 (0.55 - 1.59)	0.795	76	47	0.93 (0.54 - 1.61)	0.800
		n = 73	n = 47			n = 76	n = 47		
CXCR1 rs2854386	CC	52 (71.2)	35 (74.5)			60 (78.9)	40 (85.1)		
	CG	21 (28.8)	12 (25.5)	1.18 (0.51- 2.7)	0.697	15 (19.7)	7 (14.9)	1.43 (0.53 - 3.82)	0.771
	GG	0	0			1 (1.3)	0		
	log-Additive	-	-	-	-	76	47	1.58 (0.62 - 4.00)	0.771
		n = 73	n = 47			n = 76	n = 47		
IL-10 rs1800871	GG	22 (30.1)	19 (40.4)			25 (32.9)	19 (40.4)		
	AG	39 (53.4)	24 (51.1)	1.40 (0.63 - 3.11)	0.311	40 (52.6)	24 (51.1)	1.27 (0.58 - 2.77)	0.507
	AA	12 (16.4)	4 (8.5)	2.59 (0.72 - 9.39)		11 (4.5)	4 (8.5)	2.09 (0.58 -7.60)	
	log-Additive	73	47	1.54 (0.87 - 2.73)	0.135	76	47	1.38 (0.78 - 2.44)	0.262
		n = 73	n = 47			n = 76	n = 47		
IL-10 rs1800872	GG	23 (31.5)	20 (42.6)			25 (32.9)	21 (44.7)		
	TG	38 (52.1)	23 (48.9)	1.44 (0.65 - 3.17)	0.294	40 (52.6)	21 (44.7)	1.60 (0.73 - 3.51)	0.412
	TT	12 (16.4)	4 (8.5)	2.61 (0.72 - 9.39)		11 (14.5)	5 (10.6)	1.85 (0.55 - 6.17)	
	log-Additive	73	47	1.55 (0.88 - 2.75)	0.124	76	47	1.43 (0.82 - 2.50)	0.204

		n = 73	n = 47			n = 76	n = 47		
	CC	4 (5.5)	4 (8.5)			7 (9.2)	5 (10.64)		
IL-10 rs1800896	TC	69 (94.5)	43 (91.5)	1.6 (0.38 - 6.75)	0.520	69 (90.8)	42 (89.4)	1.17 (0.35 - 3.94)	0.796
	TT	0	0			0	0		
	log-Additive	-	-	-	-	-	-	-	-
		n = 73	n = 47			n = 76	n = 47		
	AA	42 (57.5)	24 (51.1)			43 (56.6)	21 (44.7)		
CCR5 rs1800023	AG	26 (35.6)	19 (40.4)	0.78 (0.36 - 1.70)	0.779	30 (39.5)	25 (53.2)	0.59 (0.28 - 1.23)	0.313
	GG	5 (6.8)	4 (8.5)	0.71 (0.17 - 2.92)		3 (3.9)	1 (2.1)	1.47 (0.14 - 14.95)	
	log-Additive	73	47	0.82 (0.46 - 1.45)	0.492	76	47	0.73 (0.38 - 1.39)	0.334
		n = 73	n = 47			n = 76	n = 47		
	GG	57 (78.1)	36 (76.6)			51 (67.1)	33 (70.2)		
CCR2 rs1799864	AG	12 (16.4)	10 (21.3)	0.76 (0.30 - 1.93)	0.544	24 (31.6)	14 (29.8)	1.11 (0.50 - 2.45)	0.903
	AA	4 (5.5)	1 (2.1)	2.53 (0.27 - 23.51)		1 (1.3)	0		
	log-Additive	73	47	1.07 (0.53 - 2.15)	0.849	-	-	-	-
		n = 73	n = 46			n = 76	n = 45		
	GG	20 (27.4)	12 (26.1)			18 (23.7)	11 (24.4)		
CCR5 rs1799987	AG	32 (43.8)	24 (52.2)	0.80 (0.33 - 1.95)	0.613	35 (46.1)	26 (57.8)	0.47 (0.18 - 1.21)	0.275
	AA	21 (28.8)	10 (21.7)	1.26 (0.45 - 3.56)		23 (30.3)	8 (17.8)	0.57 (0.19 - 1.71)	
	log-Additive	73	46	1.11 (0.67 - 1.85)	0.676	76	45	0.76 (0.45 - 1.30)	0.316
		n = 73	n = 47			n = 76	n = 47		
	CC	58 (79.5)	34 (72.3)			50 (65.8)	32 (68.1)		
CCR5 rs1800024	CT	11 (15.1)	11 (23.4)	0.59 (0.23 - 1.50)	0.512	25 (32.9)	15 (31.9)	1.07 (0.49 - 2.32)	1.000
	TT	4 (5.5)	2 (4.3)	1.17 (0.20 - 6.74)		1 (1.3)	0		
	log-Additive	73	47	0.83 (0.43 - 1.59)	0.569	-	-	-	-
		n = 73	n = 47			n = 76	n = 47		
	CC	26 (35.6)	17 (36.2)			24 (31.6)	15 (31.9)		
CCR5 rs1799988	CT	27 (37.0)	18 (38.3)	0.98 (0.42 - 2.30)	0.973	34 (44.7)	21 (44.7)	1.01 (0.44 - 2.35)	0.999
	TT	20 (27.4)	12 (25.5)	1.09 (0.43 - 2.79)		18 (23.7)	11 (23.4)	1.02 (0.38 - 2.75)	
	log-Additive	73	47	1.04 (0.65 - 1.66)	0.869	76	47	1.01 (0.62 - 1.65)	0.964

[a](%) number of the subjects with the specified allele or genotype.

Abbreviations: M-MICRO, women who gave birth to infants with congenital Zika syndrome; M-ZIKVexp, mothers who living in ZIKV's endemic areas who gave birth to healthy infants; C-MICRO, children with congenital Zika syndrome; C-CT, healthy children who were born of the mothers who living in ZIKV's endemic areas. Results of univariate logistic regression models
